# CD38 and Regulation of the Immune Response Cells in Cancer

**DOI:** 10.1155/2021/6630295

**Published:** 2021-02-27

**Authors:** Sanyog Dwivedi, Erika P. Rendón-Huerta, Vianney Ortiz-Navarrete, Luis F. Montaño

**Affiliations:** ^1^Laboratorio Inmunobiología, Departamento Biología Celular y Tisular, Facultad de Medicina, UNAM, Mexico; ^2^Departamento de Biomedicina Molecular, Centro de Investigación y Estudios Avanzados, IPN, Cinvestav, Mexico

## Abstract

Cancer is a leading cause of death worldwide. Understanding the functional mechanisms associated with metabolic reprogramming, which is a typical feature of cancer cells, is key to effective therapy. CD38, primarily a NAD + glycohydrolase and ADPR cyclase, is a multifunctional transmembrane protein whose abnormal overexpression in a variety of tumor types is associated with cancer progression. It is linked to VEGFR2 mediated angiogenesis and immune suppression as it favors the recruitment of suppressive immune cells like Tregs and myeloid-derived suppressor cells, thus helping immune escape. CD38 is expressed in M1 macrophages and in neutrophil and T cell-mediated immune response and is associated with IFN*γ*-mediated suppressor activity of immune responses. Targeting CD38 with anti-CD38 monoclonal antibodies in hematological malignancies has shown excellent results. Bearing that in mind, targeting CD38 in other nonhematological cancer types, especially carcinomas, which are of epithelial origin with specific anti-CD38 antibodies alone or in combination with immunomodulatory drugs, is an interesting option that deserves profound consideration.

## 1. Introduction

Cancer immunotherapy has progressed enormously since the identification of immune checkpoints. The list of putative stimulatory and inhibitory checkpoints so far clearly established is extensive: ICOS/ICOS-L, GTR/GTR-L, CD27/CD70, CD40/CD40-L, DNAM-1/CD155, MHC-II/LAG-3, PD1-L1/2, Galectin-9/TIM-3, and many more [1]. The search for new immune checkpoint targets is now centered towards the adenosinergic pathway and its metabolite adenosine (ADO), that support immune suppression within the tumor microenvironment (TME) [2, 3] as it limits the functionality of T, Dendritic, and NK cells, as well as macrophages and neutrophils [4].

The accumulation of adenosine in the TME is partly dependent on two ectoenzymes entangled in canonical and noncanonical pathways [5]. One is the ectonucleoside triphosphate diphosphohydrolase (NTPDase, CD39) that converts ATP released by cell lysis or by exocytosis of ATP-containing vesicles via transport vesicles or via lysosomes into AMP, and the other is ecto-5-nucleotidase (Ecto5¨NTase, CD73) that dephosphorylates AMP into adenosine [6]. The generation of adenosine by a noncanonical pathway begins with NAD in a reaction run by the multifunctional transmembrane protein CD38 [7].

**Figure 1:**
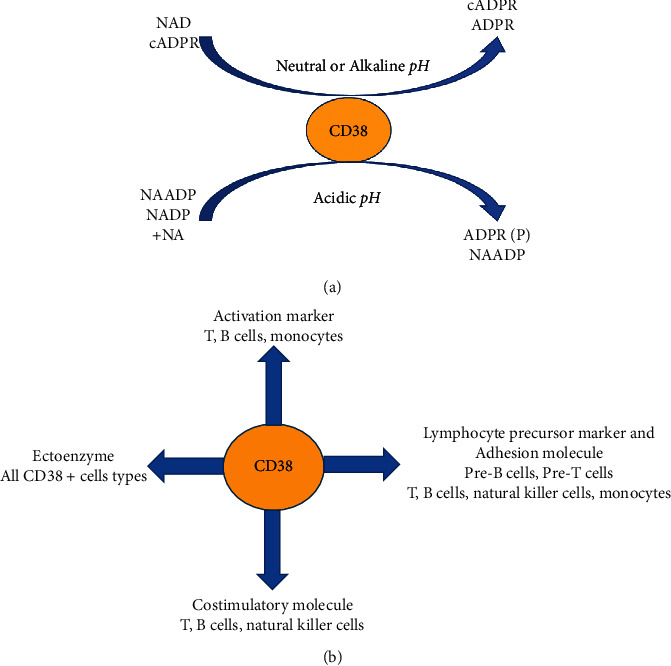
(a) Enzymatic activity of CD38 in different pH. (b) Various cell-mediated functions. Abbreviations used: cyclicADP-ribose, cADPR; ADP-ribose, ADPR; nicotinic acid adenine dinucleotide phosphate, NAADP; nicotinic acid, NA; ADP-ribose-2′-phosphate, ADPRP.

CD38 has come into consideration as its involvement in adenosine-mediated immunosuppression within the tumor microenvironment has been established [8]. CD38 is a multifunctional ectoenzyme that functions as a nicotinamide adenine dinucleotide (NAD+) glycohydrolase and catalyzes the synthesis and degradation of cADPR affecting calcium signaling and release, thus decreasing extracellular NAD+, altering calcium cascade and deeply contributing to adenosine-mediated immune suppression (Figure 1), which alters the activity of T, NK, and dendritic cells and attracts migration of suppressor cells like MDSCs, Tregs, and Bregs [9–11]. The effect upon immune cells is mainly via modulation of FasL expression [12–14]. Alterations in CD38/FasL regulated apoptosis have been reported in myeloma [15, 16] and in NK cells of gastric cancer patients [17, 18]. We have recently gathered evidence that suggests that the overexpression of some tight junction proteins in gastric cancer cells affects CD38-related FasL expression and activity on NK cells. Our aim was to emphasize the role of CD38 on the immune suppression of some malignant neoplasms and to emphasize its role as an interesting target for cancer immunotherapy [19].

### 1.1. CD38 Structure

CD38 is a 45 kDa type II transmembrane glycoprotein with a single transmembrane segment near its N-terminus. It shares a 20–30% sequence identity with Aplysia ADP-ribosyl cyclase, BST-1, also termed CD157, and a GPI-anchored protein found in *Schistosoma mansoni*. It is formed by two identical monomers that favor a physiologically stable structure with a pocket at the middle of the cleft, that is, the enzyme active site. The crystal structure of the extramembrane domain, which is fully active enzymatically and is crystallized as head-to-tail dimers, has been well determined [20, 21]. It is expressed in high densities on plasma cells, plasmablasts, natural killer cells, plasmacytoid dendritic cells, and activated B and T lymphocytes in healthy subjects and in hematological tumors including multiple myeloma [22].

### 1.2. CD 38 Function

CD38 functions as a lymphocyte receptor and transducer of signals and an ectoenzyme that generates cyclic adenosine diphosphate-ribose involved in intracellular calcium mobilization (Figure 1). First thought to be expressed only on thymocytes and activated T cells, CD38 was later found to be widely expressed on B cells, circulating monocytes, dendritic cells, granulocytes, plasma cells, both resting and circulating NK cells, neutrophils, and granulocytes. CD38 is also found on the surface of erythrocytes and platelets, where it plays an essential role, together with platelet/endothelial cell adhesion molecule 1 (CD31), in the microenvironment retention of cancer cells [22]. CD38 is also expressed in the cytoplasm and nucleus of nonlymphoid cells such as normal prostatic epithelial cells, pancreatic islet cells, smooth and striated muscle cells, renal tubules, retinal gangliar cells, and cornea.

As a surface receptor, CD38 is necessary for the activation and proliferation of immune cells. Its IFN*γ* and TNF*α* induce its expression in macrophages and dendritic cells [23]. It establishes a weak and dynamic interaction with the nonsubstrate ligand CD31, in an interaction necessary for leukocyte adhesion and migration. CD38 has a very small cytoplasmic tail suggesting it is unable to initiate a signaling cascade and so it associates with other signaling receptors such as TCR/CD3 in T cells, BCR (CD19/CD21) in B cells, and CD16/CD61 in NK cells. In addition, CD38 ligation with a counter ligand induces the expression and secretion of IL-1*β*, IL-6, IL-10, and IFN*γ* from monocytes and T cells. NAADP, produced through the enzymatic activity of CD38 [24], regulates T cell activation, proliferation, and chemotaxis.

CD38 is found in recycling endosomes that contain perforin and granzymes in the immunological synapse when the TCR of cytotoxic T cells is engaged. CD38 is expressed on membrane rafts where it promotes cell signaling via AKT and ERK activation and it is exported out of the cells through the exocytic pathway. CD38 association with the signaling complex CD16/CD61 in the NK cell membrane has a critical role in transducing activating signals. CD38^high^CD8^+^ T cells suppress the proliferation of CD38^−^CD4^+^ T cells [25], thus indicating its capacity to modulate T cell subsets with regulatory properties. CD38 signaling upon ligation induces IL-1*β*, IL-6, and IL-10 secretion and enhanced IL-12 production in synergy with IFN*γ* in dendritic cells [26, 27].

**Figure 2:**
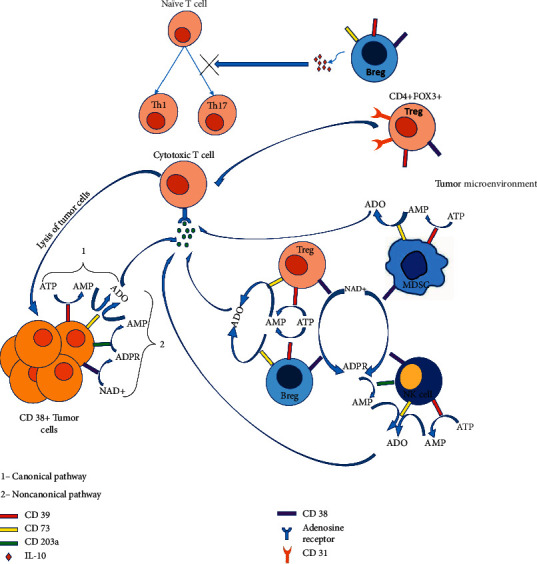
CD38 positive immune suppressive cell types; T regs, Bregs, specific Nk cell type, and tumor cells in TME produce adenosine (ADO) resulting suppression of cytotoxic activity effector T cells. CD31+ T regs contribute to immune suppression with an unknown mechanism, while Bregs prompt IL-10 mediated inhibition of naïve T cell differentiation into Th1 and Th17 but promote Treg proliferation. It has also been established that senesce drives the expression of CD38 in macrophages and endothelial cells [34]. CD38 in activated NK cells upregulates the release of IFN*γ* and TNF*α* and promotes degranulation, albeit depletion of CD38+ NK cells within the tumor does not correlate with patient response to antiCD38 treatment.

High CD38 expression in immune cells such as T regs, B regs, MDSCs, and CD16-CD56 + NK cells contribute to a change in their immune function [28–30]. A typical example of the latter is represented by the CD4+CD25^high^FOX3+ Treg cells with high CD38 expression that define a suppressive subset of Tregs in multiple myeloma and non-Hodgkin lymphoma via cytokine dependent mechanisms. However, CD31- Tregs depicted reduced immune suppressive activity that indicates the importance of CD38/CD31 interaction in Treg mediated immunosuppression [31]. CD38^high^ B reg cells produce IL-10, which inhibits T naïve cell differentiation to Th1 and Th17 cells while supporting the proliferation of T regs [32, 33]. The immunosuppressive role of myeloid-derived suppressor cells (MDSCs) is strongly expanded in the cancer microenvironment [29], which is well documented. CD38 expression is considered as a marker of MDSCs activity and CD38^high^MDSCs have more prominent immune suppressive effects. At the same time, MDSCs promote neovascularization and tumor invasion (Figure 2).

### 1.3. CD38 and the Tumor Microenvironment

Tumor microenvironment (TME), a coordinated network of immune, nonimmune, and cancer cells with other noncellular components, is vital for the development, progression, immune suppression, and persistence of cancer [35] as biological processes such as hypoxia, angiogenesis, autophagy, apoptosis resistance, and metabolic reprogramming are triggered. The enhanced concentration of adenosine in the TME leads to an increase or decrease of adenylate cyclase or intracellular cyclic adenosine monophosphate in immune cells expressing adenosine receptors (T cells, NK cells, dendritic cells, neutrophils, macrophages), thus interfering with the activation of immune cells and favoring tumor progression [36, 37]. The accumulation of adenosine within the TME causes immune suppression; targeting CD38 enzymatic activity would largely influence tumor cells. Moreover, targeting CD38 will result in an accumulation of NAD + that is by itself a danger signal.

TME is also characterized by the presence of hypoxia due to poor blood supply and increased oxygen consumption. NAD+ is produced by the salvage pathway in hypoxic TME, which is further converted to adenosine by CD38-expressing cells, thus further suppressing the immune response by recruitment of MDSCs, Tregs, tumor associated macrophages (TAMs) [38, 39]. Besides ADO arbitrated immune suppression, CD38 bestowed NAADP is also involved in VEGF mediated angiogenesis through its involvement in Ca + signaling. VEGF interacts with receptors VEGFR1 and VEGFR2. VEGF binding to VEGFR2 leads to the release of Ca++ in a process where CD38 contributes [40–43]. Therefore, cells overexpressing CD38 in the TME direct the generation of an immune suppressive environment that reduces effector T cell functions but also promotes angiogenesis provides immune escape and helps in cancer progression. For instance, in chronic lymphocytic leukemia CD38+ clones in the TME have a survival advantage over CD38- clones as they depict higher migration towards the chemokine CXCL12 resulting in enhanced homing to lymphoid tissue and improved survival with higher expression of VEGF and antiapoptotic protein Mcl1[44]. Metabolic reprogramming of NAD^+^ regulation via inhibition of CD38 has been proposed as a strategy for improving the efficacy of immune-based therapies and appears to play a significant role in the regulation of metabolism and immunomodulation of the tumor microenvironment [28, 45–47].

### 1.4. CD38 in Multiple Myeloma

Multiple myeloma is a type of cancer where malignant plasma cells overexpressing CD38 accumulate in the bone marrow. Interestingly, within the bone marrow microenvironment, myeloma cells are protected to CD38 antibody-induced cellular cytotoxicity by upregulating the expression of antiapoptotic molecules such as survivin [48]. Additionally, CD38 increases the phosphorylation of PI3K, AKT, and mTORC and upregulates the PI3K/AKT/mTOR pathway, which is related to metabolic reprogramming and proliferation of cancer cells, while such effects were significantly reversed with mTOR inhibitor, rapamycin [49, 50]. Indeed, mTOR and RICTOR are overexpressed in MM endothelial cells while mTORC2 and its downstream effectors are linked with an angiogenic switch to MM [51]. This suggests inhibition of the PI3K/AKT/mTOR pathway with a dual mTOR inhibitor along with anti-CD38 therapies will exhibit a synergistic effect in CD38-expressing MM cells [51].

In this environment, the use of anti-CD38 mAbs depletes CD38+ MDSCs, T regs, and B regs immune suppressive cells and enhances antitumor activity [52, 53], but as anti-CD38 mAbs downregulates CD38 expression in tumor cells, immune escape, and disease progression is favored [54]. Histone deacetylase (HDAC) inhibitors (Panobinostat, Ricolinostat) upregulate CD38 RNA levels and CD38 surface expression on multiple myeloma cells [55], thus disrupting latency of the malignant cells. Interestingly, an all-trans retinoic acid (ATRA) combined with anti-CD38 treatment was able to reduce complement inhibitor proteins CD55 and CD59 expression on anti-CD38 resistant cells and improved complement-mediated cytotoxicity [56]. Ricolinostat decreases phosphorylation of AKT and mTOR downstream molecules, upregulates the expression of the Th1 transcription factor T-Bet, and decreases the suppressive function of Treg cells [57]. A significant decrease in IL-10 and Foxp3 in T regs and improved proliferation and function of CD4+CD25-T cells and CD8+ and NK cytotoxic cells has been observed when an anti-CD38 monoclonal antibody was combined with lenalidomide, an immunomodulator that increases Th1 cytokine production and stimulates clonal T cell proliferation and NK cell activity [58]. STAT3, an oncogenic protein, has been targeted with a novel formulation of nanoparticles packaged with STAT 3 inhibitor linked with anti-CD38 mAb that improved uptake by MM cell lines; these nanoparticles depicted a 4-fold reduction in tumor size compared with nanoparticles carrying STAT3 inhibitor only [59].

### 1.5. CD38 in Epithelial Cancers

In esophagus cancer, tumor-derived signals such as IL-6, IGFBP3, and CXCL16 trigger the expansion of monocytic MDSCs with increased CD38 expression. This CD38+ MDSC population is halted in the early stages of differentiation, which express elevated levels of iNOS and increased activation of NFĸB, resulting to be more potent in suppressing T cells in the TME. In CD38+ cervical cancer cells, the PI3K/AKT pathway and its downstream target gene p53 expression are upregulated [49, 60]. In gastric cancer, a CD19 + CD24hiCD38hi B reg population that plays immunosuppressive roles by producing IL-10 and TGF*β* has been identified; this cell population is inhibiting cytokine production by CD4+ T cells and promotes the conversion of CD4 + CD25- effector T cells to CD4+FoxP3+Treg cells, which collectively promote tumor growth [30].

High CD38 expression has been observed in hepatic carcinoma TME and in tumor infiltrating lymphocytes (TILs). However, CD38 + TILs provide antitumor responses through secretion of cytotoxic compounds and inflammatory cytokines [61, 62]. These CD38+ TILs secrete high levels of IFN*γ* and in combination with Sorafenib, a kinase inhibitor used for advanced cancer [63], patients' survival improves notably [64]. There is a CD38+ M1 macrophage population in TME that produces high levels of IL-6 and TNF*α* with concomitant CD80 expression, prompts more inflammation and helps in tumor suppression [63]. CD38 also serves as a coreceptor in MHC-II mediated T cell activation [65]. Increased CD38 expression induces immunosuppressive effects via its adenosinergic activity and can also cause resistance to anti-PD1/PDL1 treatment in HCC patients [66]. These results reinforce, understanding variability in CD38 expression of TILs in TME may improve the effectiveness of anti-PD1 immunotherapy with a suitable anti-CD38 agent.

Studies performed with CRISPR/Cas9-based knockout of CD38 in A549 adenocarcinoma human alveolar basal epithelial cell line exhibited inhibition of anchorage-independent cell growth, cell invasion, and xenograft growth in nude mice. This is consistent with results obtained with lung cancer cell lines and patient specimens that show increased levels of CD38 mRNA and protein expression of CD38 [67]. Blocking CD38 led to a significant decrease in T regs within TME of an *in vivo* mouse model of lung cancer. CD38 is also involved in the control of nonsmall cell lung cancers (NSCLS), which cover 85% of lung cancers [68]. NSCLS treatment was centered on platinum-based chemotherapy followed by cytotoxic chemotherapy, but recently agents targeting CTLA-4 and PD-1 pathways have been included. Unfortunately, this treatment frequently develops resistance mediated by Interferon *β* and all-trans retinoic acid after a mean 5-week period, but Interestingly CD38+ CD8 T cells proliferation was induced after anti-PDL-1 therapy [69]. This CD38+ T cell population corresponds to early activated and nonexhausted effector cells [70]. The strong correlation between CD38 and inflammation in the TME supports the idea of combining anti-CD38 therapy with existing anti-PD-1/PDL-1 treatments.

Gliomas, frequent malignant brain tumors, respond poorly to conventional and recently developed cytotoxic chemotherapy. Nevertheless studies performed in CD38 deficient mice showed attenuated tumor size, progression, and improved life expectancy [71]. The glioma TME plays an important role in tumor progression [72]. Tumor associated microglia/macrophages (TMM), formed by a small proportion of resident brain CD38+ microglia and infiltrating monocytes, constitute 40% of the tumor [73, 74]. These TMMs secrete IL-1, basic fibroblast growth factor, VEGF, and regulate Ca++ mobilization via CD38 mediated cADPR, contribute to TMM activation, angiogenesis [75, 76], and immunosuppression, thus helping in tumor progression [77, 78].

In melanoma anti-PD-1 and anti-CTLA-4 based immunotherapies improve survival significantly in advanced cases [79, 80] while targeting CD38 in melanoma TME could provide synergistic effects. In mouse models, inhibition of CD38 restricted primary tumor growth and was associated with lower rates of pulmonary and brain metastasis as a result of promoted cell death, reduction in cancer-associated fibroblast, and prevention of angiogenesis. Targeting CD38 mediated NAADP synthesis, which is responsible for neoangiogenesis and Ca++ signaling, with Ned-19, an NAADP inhibitor, constrained melanoma growth, vascularization, and metastasis [42]. Thus, reinforcing the importance of CD38 mediated NAADP inhibition in the activity of tumor-promoting components of the TME. An effect of CD38 inhibition is the reduction of adenosine in the melanoma TME that results in the inhibition of antitumor responses. Fortunately the addition of adenosine in primary melanoma cell lines restores the proliferation of CD4+ and CD8+ T cells [38, 81].

### 1.6. Anti-CD38 Agents and Their Therapeutic Use

**Figure 3:**
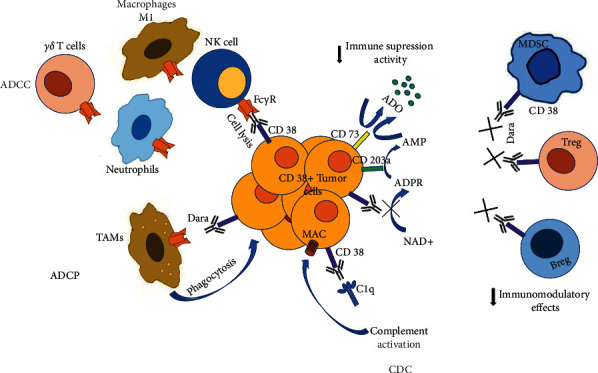
Dara mechanism of action. Dara exerts anticancer activity via Fc-dependent mechanism and immunomodulatory effects. Once bound to CD38 over cancer cells, Fc fragment of Dara allows engagement with Fc Receptors expressing effector cells, i.e., NK cells, T cells, neutrophils, and macrophages, leading to lysis (ADCC) or phagocytosis (ADCP) of the cancer cell. Engagement of Dara's Fc with C1q outcomes activation of compliment cascade and assembly of MAC complex over cancer cells and lysis (CDC). Dara´s binding mask CD38 ectoenzymatic activity reduces adenosine production and causes eliminations of immune suppressive cell types (i.e.,Tregs, Bregs, MDSCs) that promotes T cell proliferation and effector functions (the figure is reproduced from the original figure published by Overdijk et al. [84] and distributed under the Creative Commons Attribution License).

Daratumumab is a human specific IgG1 anti-CD38 approved as a single agent or in combination regimens for the treatment of relapsed/refractory multiple myeloma [82, 83]. It triggers ADCC, CDC, and TAM in CD38+ multiple myeloma cells in both sensitive and drug resistance patients, modulates the enzymatic activity of CD38, and reduces adenosine levels [84, 85]. Daratumumab combined with dexamethasone and a proteasome inhibitor (Velcade) or a TNF*α* inhibitor (Revlimid) has been approved in patients with at least one previous line of therapy. A study on newly diagnosed transplant ineligible patients compared with the gold-standard bortezomib-Melphalan-Prednisone regimen with or without Daratumumab and found that the latter increased significantly the overall response rate and improved complete response and progression-free survival [86]. A similar improvement has been determined in newly diagnosed multiple myeloma and transplant-eligible patients treated with Daratumumab and the standard Velcade-Thalidomide-Dexamethasone regime [87]. Clinical trials with Daratumumab alone or in combination with hematological and nonhematological malignancies are in progress (Table 1) (Figure 3).

Daratumumab reduces suppressive cell types in the multiple myeloma tumor microenvironment [52] as it reduces CD38 expression, but as treatment progresses, it also increases the resistance to treatment. Though this reduction is transient, it is regained in 3 to 6 months after the drug infusion. Another important concern is the reduction of CD38+ NK cells even after the first infusion. Though CD38 expression is low, NK cells retain their activity and proliferate normally [88, 89]. In such a case, reinfusion of ex vivo expanded autologous NK cells can be used.

To overcome Daratumumab mediated resistance, the use of new drugs such as a synthetic derivative of all-trans retinoic acid, Tamibarotene (a.k.a Am80), that upregulates CD38 or anti-CD38 antibodies with different mechanisms of action such as Isatuximab, Felzartamab, or Mezagitamab is recommended [90].

Isatuximab is an IgG1 monoclonal antibody that induces apoptosis of tumor cells and ADCC; it binds to a specific discontinuous epitope containing amino acids located opposite to the catalytic site of CD38, thus almost completely inhibiting cyclase activity in a dose-dependent manner [91]. Continuous Isatuximab treatment did not cause a reduction in CD38 receptor expression in H929, MM1S, OPM2, and RPMI-8226 multiple myeloma cell lines. Furthermore, Isatuximab treated cells did not show the clustering of CD38 in polar aggregates that lead to the release of CD38 in microvesicles, an effect that conduces to Daratumumab resistance [92]. Isatuximab substantiated great antitumor activity alone or in combination with dexamethasone and immunomodulatory imide drugs that include lenalidomide, pomalidomide, and iberdomide [93].

Mezagitamab (TAK-079) is a cytolytic IgG1 anti-CD38 monoclonal antibody, which effectively removes CD38+ B cell lines by antibody-dependent or complement-dependent cytotoxicity [94].

Felzartamab (MOR202) is a Human Combinatorial Antibody Library derived human IgG1 anti-CD38 monoclonal antibody that, once attached, attracts natural killer cells, triggers ADCC and ADCP but not CDC, and shows synergistically enhanced cytotoxicity with Bortezomib and Lenalidomide.

Other anti-CD38 agents are currently being evaluated. CAR-T/TCR-T, Multi-CAR-T, TAK-573, TAK-169, T-007, AMG 424, and GBR 1342 are in phase 1/2 clinical development, while others like HexaBody-CD38, CD38-ARM (KP1196, KP1237), TSK011010/CID103, STI-5171, Anti-CD38/IGF-1 R bsAb scFV, Anti-CD38 SIFbod, CAR38-MILs, CD38 DART, and Actinium-225 are in preclinical developmental stages. The significant number of potential candidates under development points to the importance of CD38 in the control of several malignancies.

Because of what has been mentioned, the efficacy of anti-CD38 antibodies in many other cancers is being evaluated in preclinical and initial stages of clinical trials (Table 1).

## 2. Conclusion

CD38 has dual functions as an ectoenzyme and as a surface receptor that promotes migratory phenotypes and signaling cascades responsible for the activation and proliferation of various immune cells.

Both canonical and noncanonical pathways contribute to adenosine synthesis. However, is targeting CD38 alone sufficient to resolve ADO induced immunosuppression? CD38 expresses ubiquitously in immune populations like T cells, NK cells, and dendritic cells; therefore, targeting CD38 would reduce anti-inflammatory response and rejuvenate antitumor activity of immune cells. But the interconnecting links between CD38, CD39, and CD73 or with downstream adenosine receptors and the persistence of any compensatory mechanism available against CD38 depletion has to be further investigated. It is clear that the enzymatic and the surface receptor functions of CD38 are distinct from each other, and there is insufficient data available to justify which function of CD38 should be targeted for effective immune function restoration and hence, tumor elimination. Nevertheless, the development of anti-CD38 monoclonal antibodies has redefined the treatment landscape due to their ability to normalize immune cells function, thus triggering antibody-dependent cell-mediated cytotoxicity, complement-mediated cytotoxicity, antibody-dependent cellular phagocytosis of opsonized CD38+ cells, and direct apoptosis via FC*γ* receptor-mediated crosslinking. As it has been clearly stated by Morandi et al.[93]: CD38 is a receptor with modulatory functions on immune regulatory cell subsets that warrants deeper analysis.

## Figures and Tables

**Table 1:** Currently active clinical trials with Daratumumab and Isatuximab.

Tumor type	Drug	Patients	Title	Reference
Carcinoma, non-small-cell lung	Daratumumab + Atezolizumab	100	A study of Daratumumab in combination with atezolizumab compared with atezolizumab alone in participants with previously treated advanced or metastatic nonsmall cell lung cancer	NCT03023423
Plasma cell myeloma	Daratumumab	28	Daratumumab in treating patients with multiple myeloma	NCT02944565
Multiple myeloma	Daratumumab	50	Study of Daratumumab in multiple myeloma (MM) patients in >VGPR/MRD-positive.	NCT03992170
Multiple myeloma	Daratumumab + dexamethasone	38	Efficacy of Daratumumab in patients with relapsed/Refractory myeloma with renal impairment	NCT03450057
Relapsed/Refractory multiple myeloma	Nivolumab-Dara/nivolumab-Dara With low dose of cyclophosphamide	62	A phase 2 study of nivolumab combined with Daratumumab with or without low-dose cyclophosphamide in relapsed/refractory multiple myeloma	
Lymphoma	Daratumumab	32	Phase 2 study to assess the clinical efficacy and safety of Daratumumab in patients with relapsed or refractory natural killer/t cell lymphoma, nasal type	NCT02927925
Relapsed/Refractory multiple myeloma	Daratumumab + ATRA	60	A phase 1 and phase 2 study of Daratumumab in combination with all-trans retinoic acid in relapsed/refractory multiple myeloma	NCT02751255
Pancreatic, non-small cell lung or triple negative breast cancers (advanced or metastatic solid tumors)	Nivolumab + Daratumumab	120	Phase 1/2 study to evaluate the safety and preliminary efficacy of nivolumab combined with Daratumumab in participants with advanced or metastatic solid tumors	NCT03098550
Smoldering plasma cell myeloma	Isatuximab	62	Phase II single arm trial of Isatuximab (SAR650984) in patients with high risk smoldering multiple myeloma	NCT02960555
Plasma cell myeloma	Isatuximab + cemiplimab	109	A phase 1/2 study to evaluate safety, pharmacokinetics and efficacy of Isatuximab in combination with cemiplimab in patients with relapsed/refractory multiple myeloma	NCT03194867
Multiple myeloma	Isatuximab + Bendamustine + prednisone	37	A phase I/II trial of Isatuximab, bendamustine, and prednisone in pentarefractory multiple myeloma	NCT04083898
Lymphoma	Isatuximab + cemiplimab	130	A phase 1/2 open-label, multicenter, safety, preliminary efficacy and pharmacokinetic (PK) study of Isatuximab in combination with other anticancer therapies in participants with lymphoma	NCT03769181
Prostate cancer non-small cell lung cancer	Isatuximab + cemiplimab	134	A phase 1/2 open-label, multicenter, safety, preliminary efficacy and pharmacokinetic (PK) study of Isatuximab (SAR650984) in combination with REGN2810, or Isatuximab alone, in patients with advanced malignancies	NCT03367819
Plasma cell myeloma	Isatuximab + lenalidomide + dexamethasone	60	A phase 1b study of SAR650984 (Anti-CD38 mAb) in combination with lenalidomide and dexamethasone for the treatment of relapsed or refractory multiple myeloma	NCT01749969
Plasma cell myeloma	Isatuximab + lenalidomide + dexamethasone	89	A phase 1b study of SAR650984 (isatuximab) in combination with pomalidomide and dexamethasone for the treatment of relapsed/refractory multiple myeloma	NCT02283775
